# Comprehensive Whole-Genome Survey and Analysis of the Naozhou Stock of Large Yellow Croakers (*Larimichthys crocea)*

**DOI:** 10.3390/ani15172498

**Published:** 2025-08-25

**Authors:** Hao-Jie Wang, Shu-Pei Huang, Eric Amenyogbe, Yue Liu, Jing-Hui Jin, Yi Lu, Charles Narteh Boateng, Zhong-Liang Wang, Jian-Sheng Huang

**Affiliations:** 1 Fishery College, Guangdong Ocean University, Zhanjiang 524025, China; wanghaojie2@stu.gdou.edu.cn (H.-J.W.); huangshupei@stu.gdou.edu.cn (S.-P.H.); ly2742706669@163.com (Y.L.); 2112201133@stu.gdou.edu.cn (J.-H.J.); hw402865@gmail.com (Y.L.); leong2006@126.com (Z.-L.W.); 2 Department of Water Resources and Aquaculture Management, University of Environment and Sustainable Development, PMB, Somanya, Eastern Region, Ghana; amenyogbeeric@yahoo.com (E.A.); cnboateng@uesd.edu.gh (C.N.B.); 3 Guangdong Provincial Key Laboratory of Aquatic Animal Disease Control and Healthy Culture, Zhanjiang 524088, China; 4 Guangdong Marine Fish Science and Technology Innovation Center, Zhanjiang 524088, China

**Keywords:** *Larimichthys crocea*, genetic diversity, aquaculture, mitochondrial DNA, breeding programs

## Abstract

The Naozhou stock of large yellow croakers (*Larimichthys crocea*) exhibits unique phenotypic traits and high genetic diversity, making it a valuable resource for selective breeding and genetic conservation in aquaculture. Despite its importance, simple sequence repeat (SSR) markers have not been developed for this stock, which limits efforts in genetic evaluation, breeding optimization, and sustainable utilization of this commercially important species. In this study, 195,263 SSRs were identified from the genome of the Naozhou stock of large yellow croaker, covering a total length of 16,578,990 bp with a density of 288 bp/Mb. Dinucleotide repeats were the most common, with the AC motif being the most prevalent. The frequency of SSR markers ranged from 245.63 to 346.60 per Mb. A total of 30 primer pairs were synthesized, of which 28 pairs (93.3%) successfully amplified clear and reproducible bands in PCR assays. Among these, 28 SSR markers exhibited distinct and reproducible bands following gel electrophoresis. For eight SSR loci, the number of alleles (Na) ranged from 4 to 22 (mean = 11.375), while the effective number of alleles (Ne) ranged from 1.5401 to 10.4727 (mean = 5.6475). The assembled mitochondrial genome (mtDNA) was 16,467 bp in length and comprised 37 genes, including 13 protein-coding genes (PCGs), 22 tRNA genes, and 2 rRNA genes. The total sequence length of the PCGs was 11,431 bp, accounting for 69.4% of the mtDNA. A large portion of the PCGs (5) used incomplete stop codons (e.g., *nad2*, *nad3*, *cox2*), while others used TAA stop codons (e.g., *nad6*, *nad5*, *TrnT*). The mtDNA encoded a total of 3808 codons, with UAA showing the highest relative synonymous codon usage value. The SSR markers and mtDNA data generated in this study provide valuable tools for future genetic breeding and genomic research on the Naozhou stock of large yellow croakers.

## Simple Summary

The large yellow croaker is a popular marine fish in China, especially valued for its taste and commercial importance. One particular group, known as the Naozhou stock, has unique features that make it ideal for fish farming, such as better meat quality, strong stress resistance, and delayed maturity. However, efforts to breed and protect this stock have been limited by a lack of genetic information. This study used modern DNA sequencing to uncover important genetic data about this fish, including its full mitochondrial genome and over 195,000 genetic markers called microsatellites. These markers help scientists understand the genetic diversity of the fish and can guide the selection of better traits for breeding. The results showed that the Naozhou stock has high genetic diversity, making it a valuable resource for improving aquaculture. The genetic tools developed in this study will help in tracking fish breeding, conserving unique populations, and improving fish farming practices. This work provides a foundation for sustainable aquaculture and conservation of this important marine species, contributing to food security and the future of environmentally responsible fish farming.

## 1. Introduction

The large yellow croaker (*Larimichthys crocea*), a member of the Sciaenidae family, is primarily distributed in the southern Yellow, East, and northern South Seas in China [1,2,3,4]. Valued for its delicate flesh, this species has become a highly sought-after food fish and is one of the most economically important marine fish in China [5,6,7]. Previous studies have classified *L. crocea* into three distinct geographical stocks: the Dai-qu stock (from the southern Yellow Sea to the central East Sea), the Min-Yuedong stock (from the southeastern East Sea to the northern South Sea), and the Naozhou stock (from the west of the Pearl River Estuary to the Qiongzhou Strait in the South Sea) [8,9,10]. The Naozhou stock exhibits distinct phenotypic characteristics that set it apart from the eastern coastal stocks and retains many traits typical of wild populations, demonstrating a richer genetic diversity. Compared with other stocks, individuals from the Naozhou stock show slower yet more stable growth rates, which are advantageous for selective breeding under intensive aquaculture conditions. Their deeper body coloration and brighter skin are highly favored in the consumer market, contributing to increased commercial value. Moreover, this stock demonstrates enhanced tolerance to environmental stressors such as temperature fluctuations and low dissolved oxygen, which is likely attributable to its preservation of wild-type genetic diversity characteristics. In addition, the Naozhou stock possesses thicker muscle fibers and a firmer flesh texture, which improve sensory quality [1,2,3,7]. Delayed sexual maturation has also been observed, potentially extending the growth period before spawning and enhancing feed conversion efficiency. These characteristics suggest that the Naozhou stock holds valuable characteristics often lost in intensively farmed populations. Its preservation could play a key role in selective breeding programs aimed at enhancing resilience, flesh quality, and adaptability to variable marine environments. However, the Naozhou stock has experienced a marked decline in population size. Therefore, developing a breeding program that enhances traits suitable for deep sea and offshore aquaculture is essential for advancing the large yellow croaker farming industry and expanding it into more complex marine environments.

Microsatellites, also referred to as simple sequence repeats (SSRs) or short tandem repeats (STRs), are DNA sequences composed of tandemly repeated units of 2–6 base pairs (bp), flanked by unique but conserved sequences within populations [11]. Owing to their abundance, polymorphism, co-dominance, strong repeatability, and widespread distribution throughout the genome, microsatellites have become valuable molecular markers [12]. They are widely applied in population genetics, phylogenetic analysis, germplasm identification, genotyping, and the construction of genetic linkage maps, particularly in aquatic species [13]. Despite their utility, research on microsatellites in large yellow croakers remains limited. The lack of genomic research has impeded the effective management and utilization of this species’ genetic diversity. Consequently, the identification and characterization of additional highly polymorphic and stable microsatellite loci from the *L. crocea* genome are urgently needed. Such markers contribute to the genetic improvement and conservation in the large yellow croaker aquaculture industry.

A genome encompasses both functional and non-functional DNA sequences that define an organism’s biological identity [14]. In recent decades, the advancement of high-throughput sequencing technologies has accelerated genome sequencing efforts across taxa [15]. As of December 2019, approximately 270 fish genomes had been assembled and made publicly available through the NCBI Genome database, supporting research in comparative genomics, systematics, and aquaculture. With over 34,000 fish species recorded in FishBase, large-scale initiatives like the Earth BioGenome Project are making comprehensive genome sequencing of fish species increasingly feasible, enabling deeper insights into their biology, evolution, and utility in sustainable fisheries and aquaculture [16,17,18,19,20].

Genome-wide survey sequencing (GSS), based on high-throughput sequencing technology, offers a rapid and efficient approach for generating a global perspective for high-quality genome assembly. It also serves as a fundamental tool for low-depth sequencing in non-model species that lack reference genomes [7,21,22]. In aquaculture genomics, the identification of genetic determinants of key production and performance traits is central to advancing selective breeding programs. This field has been widely discussed in review papers [23,24], conference proceedings [25,26], and books [18,27]. Whole-genome sequencing (WGS) has been widely applied to a variety of aquatic fish species, including the *Cyprinus carpio* [28], *Platycephalus* sp.1 [29], *Muraenolepis orangiensis* [30], *Paralichthys orbignyanus* [31], *Pampus* spp. [32], and *Chionobathyscus dewitti* [33]. WGS enables the characterization of essential genomic features such as genome size, heterozygosity levels, repeat sequence content, and guanine–cytosine (GC) content. Additionally, the resulting genomic data support the development of genome-wide microsatellite (SSR) markers and the assembly of mitochondrial genome (mtDNA), as demonstrated in *Platycephalus* sp.1 and *Acanthocepola indica* [34].

The mtDNA is a circular, double-stranded molecule typically composed of 13 protein-coding genes (PCGs), 22 tRNA genes, 2 rRNA genes, and a control region [35,36,37]. However, genomic and mtDNA information specific to the Naozhou yellow croaker remains unavailable. To date, only nucleotide sequences from the Dai-qu and Min-Yuedong stocks are available in the GenBank database (www.ncbi.nlm.nih.gov/genbank/) (accessed on 18 December 2024). This lack of genomic resources limits the implementation of effective genetic breeding strategies and conservation efforts for the Naozhou stock of large yellow croakers. Therefore, a comprehensive genomic investigation of this stock is essential for expanding the genetic resource database of the species, facilitating marker-assisted breeding and optimizing aquaculture.

In this study, we conducted the first GSS of the Naozhou yellow croaker using DNBseq technology. Key genomic features, including genome size, GC content, and heterozygosity, were estimated and analyzed. Additionally, genome-wide SSRs were identified and applied to assess the population structure across two *L. crocea* stocks. The mtDNA of the Naozhou stock was also assembled, and its PCGs were analyzed. These genomic resources provide valuable data for future studies on the genetic breeding and population genetics of the Naozhou stock of *L. crocea*.

## 2. Materials and Methods

### 2.1. Sample Collection

**Figure 1:**
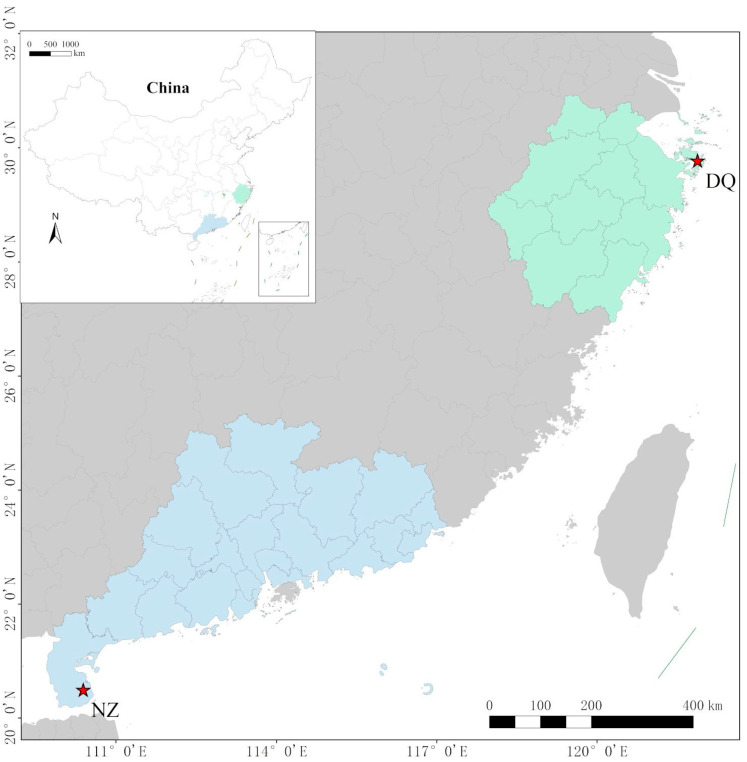
Geographic location map of the sampling points.

In this study, a male yellow croaker was collected near Naozhou Island in the wild, Zhanjiang, Guangdong Province, China (20°41.404′ N, 110°34.547′ E) (Figure 1). The specimen weighed 249.6 g and measured 27.3 cm in length. After anesthesia with 100 mg/L MS-222, muscle tissue samples were collected, immediately frozen in liquid nitrogen, and subsequently stored at −80 °C. Genomic DNA was extracted using the phenol–chloroform method [38]. DNA quantification was performed using a NanoDrop 2000 spectrophotometer (Thermo Fisher Scientific, Waltham, MA, USA), and DNA integrity was evaluated by 1.2% agarose gel electrophoresis. The sampling procedure was approved by the Animal Ethics and Use Committee of Guangdong Ocean University (GDOU-LAE-2023-054).

### 2.2. Genome Sequencing and Assembly

High-quality DNA was randomly fragmented into 300–400 bp fragments using a Covaris ultrasonicator (Covaris, Woburn, MA, USA), following the manufacturer’s protocol. A sequencing library was constructed through end-repair, A-tailing, adapter ligation, purification, and Polymerase Chain Reaction (PCR) amplification. Paired-end sequencing (PE150) was performed using the DNBseq platform at the Beijing Genomics Institute, China, to generate high-throughput sequencing data.

Raw reads were processed using Fastp v1.0.1 [39,40,41] to remove adapter sequences, low-quality reads, and duplicates. The filtered high-quality reads were de novo assembled into contigs and scaffolds using SOAPdenovo v 2.03 [42,43] with the following parameters: “-K53 -R -M3 -d1”. All raw sequencing data were freely available in the Sequence Read Archive (SRA) database (http://www.ncbi.nlm.nih.gov/sra/) (accessed on 5 January 2025) under the accession number PRJNA1169539.

### 2.3. K-Mer Analysis

Genome size, heterozygosity, and GC content of marbled rockfish (*Sebastiscus marmoratus*) were estimated using K-mer analysis (K = 21) [44,45,46] and GenomeScope 2.0 [47,48]. First, the sequencing data were processed to generate a K-mer depth distribution, and the K-mer frequency spectrum was fitted to assess the level of heterozygosity and the proportion of repetitive sequences in the genome. Second, a scatter plot based on GC distribution and sequencing depth was constructed to visualize the genome characteristics.

### 2.4. Microsatellite Mining and Primer Design

Microsatellite sites in the entire genome were identified using MISA software v2.1 (http://pgrc.ipk-gatersleben.de/misa/) (accessed on 5 January 2025) [49,50]. The length of the repetitive sequence was defined as the default range of single nucleotide to hexanucleotide. The search parameters were set as follows: the number of single base repeats was 10 or more; the number of 2 base repeats was 6 or more; and the number of 3–6 base repeats was 5 or more. If the interval between two microsatellites was less than or equal to 100 bp, they were considered to form a composite microsatellite [51,52]. The primer selection parameters were as follows: a final product length of 80–500 bp, a primer length of 20–28 bp, GC content of 40–60%, and a primer melting temperature of 60–65 °C.

### 2.5. Microsatellite Verification and Genetic Diversity Analysis

To further analyze the microsatellite distribution characteristics, 30 primer pairs were selected and synthesized by Sangon Biotech (Shanghai, China) (Table S1). The PCR system included 12.5 μL of 2 × PCRMix, 0.5 μL each of forward and reverse primers (10 μmol/L), 1 μL of DNA template (10–50 ng), and 10.5 μL of double distilled water (ddH2O). The reaction conditions were as follows: 95 °C for 5 min, 95 °C for 30 s, 52–62 °C for 30 s (according to the Tm value corresponding to the primer), 72 °C for 30 s for 25 cycles, and finally, extension at 72 °C for 5 min. The PCR products were tested for the success rate of sample amplification by 2% (M/V) agarose gel electrophoresis. Markers that produced clear bands but showed nonspecific amplification were optimized to reduce nonspecific amplification. Subsequently, the target microsatellite loci were PCR amplified, and the amplified products were first checked by 1% agarose gel electrophoresis. Primers that produced expected sizes were selected for further analysis. Forward primers were fluorescently labeled with either hexachlorofluorescein or 6-carboxyfluorescein at the 5′ end. After fluorescent PCR amplification, the products were subjected to capillary electrophoresis using an ABI 3730XL system (Applied Biosystems, Foster City, CA, USA), and the output data were analyzed using GeneMapper v 6.0 (www.thermofisher.cn/order/catalog/product/4475073?SID=fr-cesoftware-1/) (accessed on 10 January 2025). Eight polymorphic SSR loci were randomly selected (Table S1) to assess the genetic diversity of large yellow croakers from the Naozhou and Dai-qu stocks. The number of alleles (Na), effective number of alleles (Ne), observed heterozygosity (Ho), expected heterozygosity (He), and Shannon information index (I) were calculated using Popgene v1.32 (https://sites.ualberta.ca/~fyeh/popgene_download.html/) (accessed on 10 January 2025) The polymorphism information content (PIC) was calculated using Cervus v 3.0.7 [44]. To investigate the distribution characteristics of microsatellites, thirty individuals of the Naozhou stock of *L. crocea* were used for marker validation. Additionally, thirty individuals from both the Naozhou and Daiqu stocks were employed to assess genetic diversity.

### 2.6. Assembly and Annotation of the mtDNA

To obtain clean data, the WGS data were aligned to the reference sequence using minimap2, Minimap2-2.30 (r1287) [30,53], and the sequences were then assembled using SPAdes v 3.15.4 [54,55] with default parameters. Bandage v 0.8.1 software was used to visualize and adjust the assembly results and to obtain preliminary results [56]. After exporting the assembled sequences, redundant sequences were removed, and the starting point of the sequences was adjusted according to the reference sequence. Mitos2 [33] was used to obtain preliminary results, which were then aligned to the reference sequence and manually corrected. The circle diagram was drawn using OGDraw software version 1.3.1 [57].

## 3. Results

### 3.1. Sequencing Data Statistics and Quality Assessment

A total of 66.81 Gb of raw data was generated using the DNBseq platform. After filtering and quality assessment, about 65.39 Gb of high-quality clean data were obtained, with Q20 and Q30 scores of 98.14% and 93.96%, respectively (Table 1). The base frequency distribution showed no AT or GC separation and no mixed base N under paired-end sequencing. The average GC content was 41.47%, displaying a unimodal distribution. The results showed no apparent exogenous DNA contamination. The above results further proved that the sequencing data met the quality and quantity requirements for subsequent analysis.

### 3.2. K-Mer Analysis and Estimation of Genome Size and Heterozygosity

**Figure 2:**
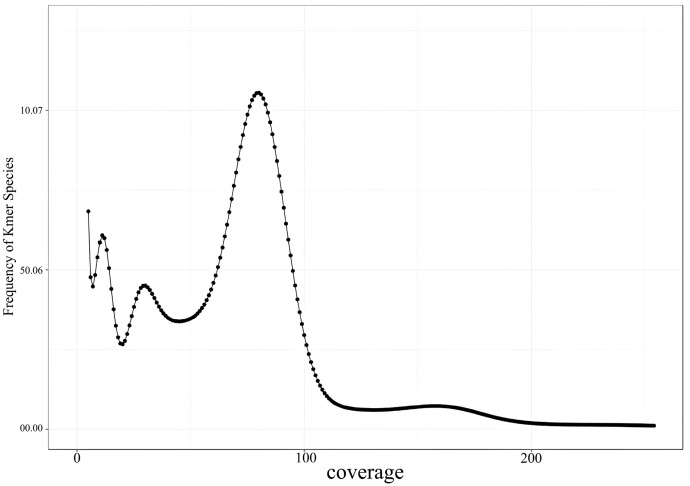
K-mer analysis for the genome size estimation of the Naozhou stock large yellow croaker. The horizontal axis represents the K-mer depth, and the vertical axis represents the frequency of the corresponding depth.

**Figure 3:**
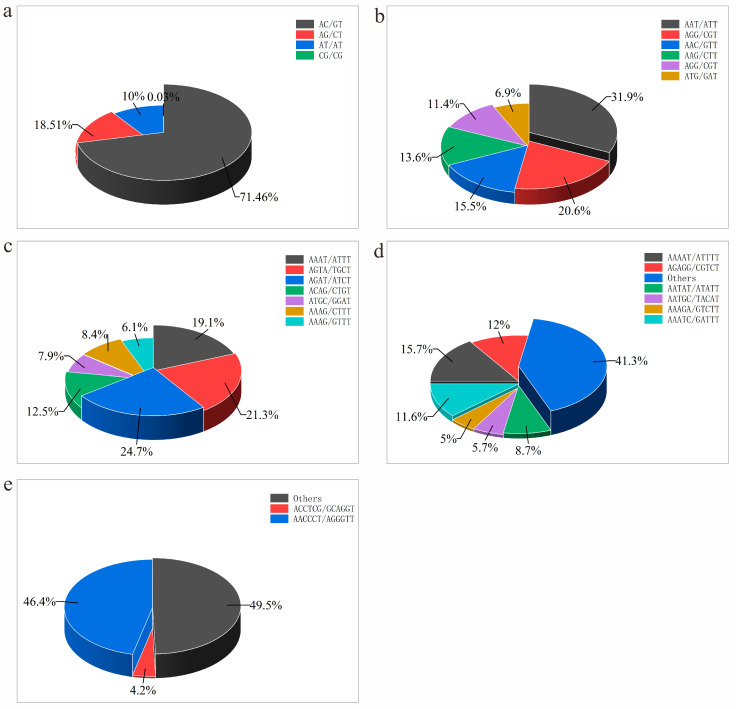
Distribution of microsatellite gene sequences in the Naozhou stock large yellow croaker. (**a**) Distribution of dinucleotide repeat SSRs. (**b**) Distribution of trinucleotide repeat SSRs. (**c**) Distribution of tetranucleotide repeat SSRs. (**d**) Distribution of pentanucleotide repeat SSRs. (**e**) Distribution of hexanucleotide repeat SSRs.

The genome characteristics of the Naozhou yellow croaker were estimated using K-mer analysis with K = 21 (Figure 2). The genome size was estimated to be 677.78 Mb, with a heterozygosity rate of 0.839% and a repeat sequence ratio of 22.181% (Table 2). No heterozygosity peak was observed in the K-mer distribution scatter plot (Figure 3).

The assembly results are shown in Table 3. The N50 length was 29.07 Mb, and the longest sequence length reached 34.52 Mb. Paired-end sequencing reads were aligned to the initial contigs to facilitate genome map construction. By merging and removing branches and bubbles, we realigned the reads to the contigs and connected the contigs into scaffolds using paired-end information. Statistical analysis showed that the N50 length of the scaffold was 1299 bp and the longest sequence length was 58,869 bp.

### 3.3. Analysis of Genomic Microsatellite Loci

MISA software v2.1 was used to screen and identify microsatellite markers in the assembled genome sequence of the Naozhou large yellow croaker. A total of 195,263 microsatellite loci were detected, with a relative abundance of 288/Mb, corresponding to one locus every 3.47 Kb on average. The total length of all microsatellite sequences was 1,6578,990 bp, with an average length of 39.59 bp. The composition type and number of microsatellites were analyzed, and the results showed that there were 73,009 composite microsatellite loci, accounting for 37.39% of all SSRs. Dibasic repeats were the most abundant microsatellites in the genome, comprising 73.8% (150,533/345,796) of the total microsatellites. The remaining repeat types accounted for a very low proportion, with 17.93% (35,019/345,796) being trinucleotides, 5.9% tetranucleotides, 1.94% pentanucleotides, and 0.36% hexanucleotides. These six types of repeats accounted for only 26.2% of the total microsatellites (Table 4).

**Figure 4:**
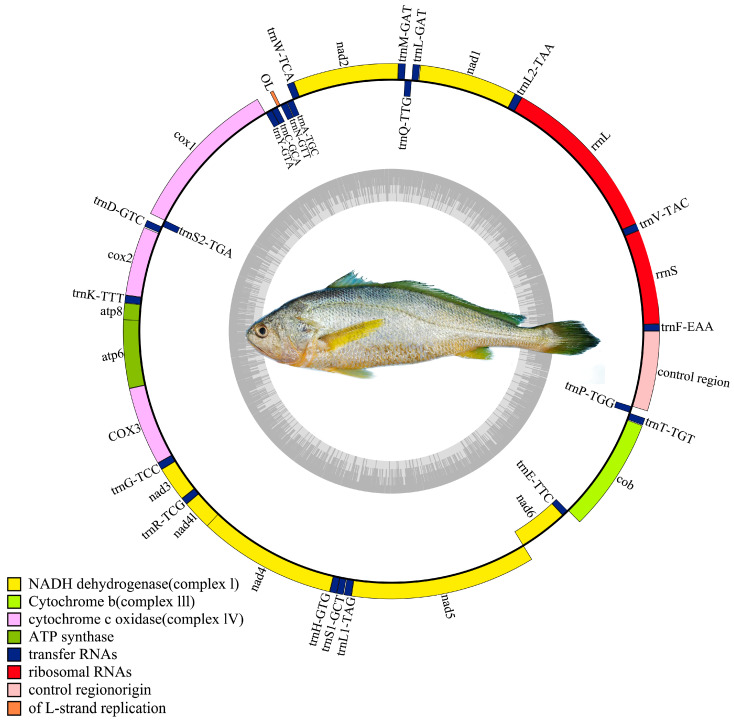
Mitochondrial genome assembly of the Naozhou stock of large yellow croakers.

A 3-D bar graph was drawn based on the number of repeats and SSRs. A total of 244 SSR types were detected, and the most abundant SSR category was the (AC)n repeats (142.34 sites/Mb). The second and third most abundant SSRs were (AG)n (26685, accounting for 36.85 sites/Mb) and (AT)n (14421, accounting for 1.92 sites/Mb), respectively (Table 5; Figure 4). Among the dinucleotide repeats, (CG)n had the lowest number, with only 45 sites and a density of 0.06 sites/Mb. For trinucleotide repeats, AAT/ATT repeats were the most abundant (10159, with a frequency of 14.03 sites/Mb), followed by AGG/CTT (6571, with a frequency of 9.07 sites/Mb). Although tetranucleotide, pentanucleotide, and hexanucleotide SSR loci contained more pattern types, their respective proportions were relatively low (Table 5). AGAT/ATCT, AAAAT/ATTTT, and AACCCT/AGGGTT were the major SSR patterns in tetranucleotides, pentanucleotides, and hexanucleotides, respectively.

### 3.4. Microsatellite Marker Verification and Diversity Analysis

To evaluate the polymorphism of the identified SSR markers, 30 primer pairs were synthesized (Table S1). Among these, 28 pairs (93.3%) produced clear and reproducible PCR amplification bands. The remaining two primer pairs either failed to show polymorphism or produced no amplification products. Several primers also showed nonspecific amplification and/or blurred bands. Eight primer pairs were selected and analyzed by capillary fluorescence electrophoresis in two stocks of the large yellow croaker.

Genetic diversity in the Naozhou large yellow croaker was assessed using the eight SSR loci (Table 6). The Na ranged from 4 to 22, with an average of 11. The Ne ranged from 1.5 to 10, with an average of 5.6. The Ho and He varied from 0 to 0.88 (average: 0.33) and from 0.36 to 0.92 (average: 0.76), respectively, while I ranged from 0.27 to 2.5, with an average of 0.68. In comparison, the Dai-qu large yellow croaker exhibited Na values ranging from 3 to 15 and Ne values from 1 to 10 (Table 7). Similarly, the average Ho and He values ranged from 0.0833 to 1 and 0.15 to 0.92, respectively (Table 7). These results suggest that the genetic diversity of the Naozhou stock is higher than that of the Dai-qu stock.

### 3.5. MtDNA Assembly

The mtDNA of the Naozhou large yellow croaker was assembled and annotated as a closed circular molecule of 16,467 bp, comprising 39 genes, including 13 PCGs, 22 tRNA genes, and 2 rRNA genes (Figure 4). Among these, 9 genes (*trnQ*, *trnA*, *trnN*, *trnC*, *trnY*, *trnS2*, *nad6*, *trnE*, and *trnP*) were located on the light strand (L-strand), while the remaining 30 genes were positioned on the heavy strand (H strand). The overall GC content of the mtDNA was 46.93%.

The 13 PCGs consisted of 7 NADH dehydrogenase genes, 3 cytochrome c oxidase genes, 2 ATP synthase genes, and 1 cytochrome b gene, which were used to calculate the relative synonymous codon usage analysis. Notably, five PCGs (*nad2*, *nad3*, *cox2*, *cox3*, and *nad4*) terminated with incomplete stop codons. Among the remaining genes, *nad6*, *nad5*, *cob*, *nad4l*, *atp8*, and *atp6* terminated with TAA, while *cox1* and *nad1* terminated with AGA and TAG, respectively.

**Figure 5:**
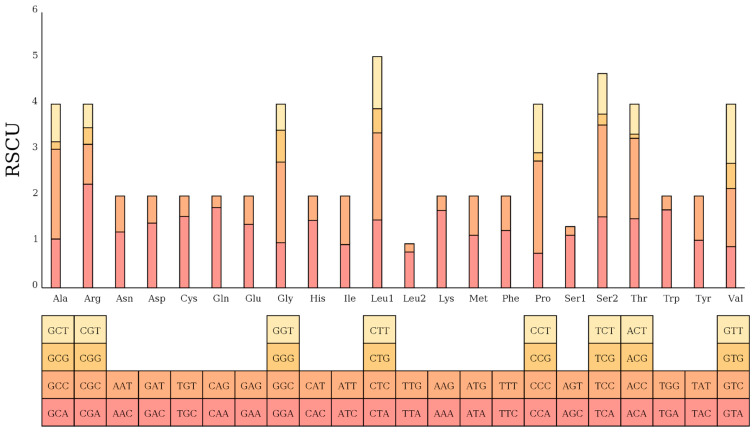
Amino acid preference for codons.

Most amino acids exhibited a preference for specific codons, such as CAA (Gln), CAC (His), and CCC (Pro). Additionally, amino acids including Pro, Thr, Leu, Ala, Ser, Val, and Gly exhibited relatively high frequencies (>5%), likely due to being encoded by four or six codons. However, exceptions were observed, with Arg comprising only 2.09% of the total amino acids in the PCGs. In contrast, Ile and Phe, each encoded by only two codons, accounted for 7.1% and 6.7%, respectively. These findings provide insights into codon usage bias and amino acid composition in the mitochondrial PCGs (Figure 5).

## 4. Discussion

The advancement of next-generation sequencing technologies has made it more accessible for researchers to explore a wide range of genome-related biological questions, particularly in non-model species [44,58]. WGS data enable the estimation of key genomic characteristics, including genome size, heterozygosity ratio, and repeat ratio, using bioinformatics approaches without requiring prior knowledge [59]. The comprehensive whole-genome survey and analysis of the Naozhou stock of large yellow croakers have provided valuable insights into its genomic characteristics, genetic diversity, and potential applications in aquaculture optimization and genetic conservation. This study successfully identified genome-wide SSR markers and assembled the mtDNA, which are critical for genetic evaluation, selective breeding, and conservation. In recent years, microsatellite markers have gained widespread application across various fields, including studies of genetic diversity [60,61], marker-assisted breeding [62], gene mapping, and quantitative trait loci (QTL) analysis [3,28,63,64].

The K-mer analysis conducted in this study revealed the genome size of the Naozhou large yellow croaker to be approximately 677.78 Mb, which is smaller than that of other marine fish species, including the *C. dewitti* (880 Mb) [33], *Morone saxatilis* (797 Mb) [65] *Anthias nicholsi* (815 Mb) (Liu et al., 2024) [66], and *Clupea harengus* (850 Mb) [67] but similar to that of *Sardina pilchardus* (625–637 Mb) [68] and *Trachinotus ovatus* (642.68 Mb) [36] The size and variability of eukaryotic genomes are influenced by various factors, including mutation pressure, transposon activity, genome ploidy, biological life history traits, and environmental conditions [69,70]. Larger genomes are generally associated with longer evolutionary histories and a higher risk of extinction [71]. The repeat sequence proportion in the genome of the Naozhou large yellow croaker was 22.181%, which was considered medium-low, lower than that of the *Chiloscyllium plagiosum* (63.53%) [72], *Hemitripterus villosus* (38.61%) [73], and *Trachinotus carolinus* (30.19%) [74], but similar to the *Ameiurus nebulosus* (39.65%) [44], *M. saxatilis* (39.22%) [65], and *A. nicholsi* (39.69%) [66]. The observed genome heterozygosity and repeated content suggest that the Naozhou large yellow croaker retains genetic traits that may confer advantages for adaptation and resilience in natural and aquaculture environments. These results suggest that the Naozhou stock exhibits moderate genome complexity, with a relatively lower repeat sequence proportion compared to other marine fish species.

The proportion of repeat sequences in a genome is crucial for designing genome sequencing strategies, as it facilitates the selection of appropriate genome assembly methods. The GC content in most fish species typically ranges from 40% to 46% [75,76,77]. For the large yellow croaker, the heterozygosity rate was 0.839%, with a GC content of 41.47%. This value is lower than that of the *Hemitripterus villosus* (43.13%) [73] and the icefish *C. dewitti* (49.9%) [33], but comparable to that of the *sebastiscus marmoratus* (41.3%) [44], *Acanthogobius omaturus* (40.88%) [76], and *Acanthopagrus latus* (42.07%) [78]. The GC content of 41.47% falls within the typical range for fish species (40–46%), indicating a stable genome composition. Additionally, the absence of contamination and high sequence quality (Q20: 98.14%; Q30: 93.96%) ensures the reliability of subsequent analyses.

This study identifies a high-density panel of 195,263 SSR markers in the Naozhou stock genome, providing a valuable resource for genetic applications. The relative abundance (288 loci/Mb) and high polymorphism rate, particularly of AC/GT dinucleotide repeats, are comparable to or exceed those reported in related species such as *Harpadon nehereus*, *Synbranchus marmoratus*, *Gadus macrocephalus*, *Pogonophryne albipinna*, *Siganus oramin*, and *Acanthogobius ommaturus* [23,29,75,76,79,80,81]. These SSRs, especially the 28 primer pairs with a high amplification success rate (93.3%), provide a solid foundation for constructing high-resolution genetic maps, which are essential for marker-assisted selection (MAS) in aquaculture [23]. This genome-wide SSR development marks a considerable improvement over earlier studies that employed traditional methods for SSR identification, typically relying on expressed sequence tag (EST) libraries or limited genomic libraries. For example, previous works such as Zhang et al. [3] employed mitochondrial COI sequences to study population structure, which, while useful for phylogeographic inference, lacked the resolution and co-dominant inheritance pattern of SSRs. Additionally, the Naozhou stock exhibited high genetic diversity (Na = 4–22; He up to 0.9238), exceeding that of the Dai-qu stock. This suggests strong adaptive potential and resilience, which are vital for selective breeding under diverse aquaculture conditions. The PIC values, averaging 0.718, indicate a robust capacity to discriminate between individuals, a prerequisite for effective parentage analysis, QTL mapping, and population structure assessments [45,82]. In Chen’s study on the heat resistance of large yellow croakers (*L. crocea*), all three microsatellite markers associated with thermal tolerance contained AC as their repeat motifs, which is consistent with the findings of the present study (thermal tolerance evaluation and related microsatellite marker screening and identification in the large yellow croaker (*L. crocea*)). With increasing fishing pressure, Wang et al. conducted a microsatellite analysis of both wild and cultured populations of *L. crocea* and found that the cultured populations exhibited lower genetic diversity compared to wild populations, further underscoring the importance of conserving wild genetic resources (loss of genetic diversity in the cultured stocks of the large yellow croaker, *L. crocea*, revealed by microsate).

The high-frequency AC/GT motifs are not merely statistical artifacts; these motifs are associated with regulatory regions involved in gene expression and chromatin structure [38]. Their prevalence may indicate genomic regions of evolutionary and functional importance in *L. crocea*, particularly under environmental pressures such as salinity and temperature fluctuations common in offshore farming. In comparison to other marine teleosts, the SSR composition of the Naozhou genome aligns with broader trends in fish genomics but also reveals stock-specific signatures. For example, while trinucleotide repeats dominate in species such as *Dicentrarchus labrax*, *Salmo salar*, and *Takifugu rubripes*, the Naozhou stock is characterized by a predominance of dinucleotide SSRs. This divergence may reflect distinct evolutionary histories and selection pressures, potentially linked to ecological niches or demographic events [83]. By establishing a dense SSR marker panel for the previously under-studied Naozhou stock, this study addresses a critical gap in marine aquaculture. These markers not only function as genetic barcodes but also facilitate lineage tracking, inbreeding monitoring, and adaptive capacity assessment. This directly supports long-term sustainability in breeding programs and biodiversity conservation [25,84].

Moreover, microsatellite loci from earlier studies were often limited in number and polymorphism, with reported allele numbers typically ranging from 3 to 8 [3,85]. Furthermore, while genome-wide approaches have been used to examine stress adaptation in *L. crocea*, such as in Ao et al. [86], who explored the molecular responses to hypoxia and thermal stress, these studies did not focus on the systematic development of polymorphic SSRs. Similarly, Xu et al. [87] characterized the hsp70 gene family under cold and heat stress conditions but did not provide transferable genetic markers for population studies or breeding programs. The SSR markers developed in this study are directly representative of this wild-type diversity and thus have high relevance for selective breeding, conservation, and population structure monitoring in both hatchery and natural settings. Additionally, the newly developed SSRs are positioned to overcome the limitations of earlier markers. For instance, SSRs used in population differentiation studies like those by Zhang et al. [3] and Kon et al. [85] often suffered from poor genome coverage and limited polymorphism, which constrained their ability to capture fine-scale genetic structure or support genome-wide association studies (GWASs). In contrast, the current study provides a genome-wide inventory with comprehensive coverage, higher repeat motif diversity (dominated by AC dinucleotides), and validated primer sets that exhibit strong amplification and polymorphic potential. To further enhance the contribution of this study, the newly developed SSR loci should be compared against existing published SSR datasets in future study. Metrics such as polymorphism information content (PIC), expected heterozygosity (He), and allelic diversity should be evaluated to determine the relative informativeness and transferability across other geographical stocks, including the Dai-qu and Min-Yuedong populations [3,85,88]. The genome-wide SSR development for the Naozhou stock of *L. crocea* provides a rich molecular resource with higher resolution and broader applicability than prior microsatellite datasets. It represents a critical step toward the genetic improvement, conservation, and management of this economically significant species. Comparing these new loci with existing SSR panels will further consolidate their utility in nationwide aquaculture genetics and breeding strategies.

The assembled mtDNA sequence of the Naozhou large yellow croaker was 16,467 bp in length, consistent with the Dai-qu stock of large yellow croakers (PRJNA927338). The genome adheres to the canonical gene order observed in teleosts, comprising 13 PCGs, 22 tRNAs, and two rRNAs, providing confidence in the completeness and integrity of the assembly [29,61,89]. Most PCGs initiate with the ATG codon, and several utilize incomplete stop codons (e.g., T) that are post-transcriptionally completed, consistent with mechanisms seen in other *L. crocea* stocks [8] and marine fishes generally [90]. The overall mtDNA structure closely mirrors that of the Dai-qu stock, with minor variations in the control region, a recognized hotspot for polymorphism and regulatory evolution [35]. Codon usage exhibits a bias toward UAA and codons encoding Proline, Threonine, and Leucine, suggesting functional constraints and possible translational optimization. Codon bias and AT richness (overall GC content of 46.93%) in the mtDNA may influence mitochondrial gene expression, thereby impacting traits like energy metabolism, stress tolerance, and growth performance. These characteristics are critical for aquaculture productivity, particularly under variable environmental conditions [91]. This study’s dual contributions, namely the development of high-density SSR marker and full mtDNA assembly, substantially enhance genomic resources for *L. crocea*, especially for the genetically distinct and underutilized Naozhou stock. The genetic differentiation from other stocks highlights the necessity of implementing stock-specific breeding programs to maintain genetic integrity and avoid homogenization [7]. These results enable fine-scale genetic mapping and MAS for traits such as disease resistance and growth. Additionally, they support population genomic studies to monitor stock integrity and inbreeding, enable comparative genomic studies with other marine species for evolutionary and functional analyses, and contribute to conservation strategies for declining stocks. By integrating both nuclear (SSR) and mtDNA genomic insights, this study provides a comprehensive genomic toolkit that can guide future transcriptomic, epigenetic, and functional validation studies [14,18]. The development of genome-wide SSR markers and mtDNA assembly in this study significantly advances current knowledge of the Naozhou stock of *L. crocea*. It marks a transition from mere genomic description to providing actionable insights for functional studies, evolutionary biology, and sustainable aquaculture. These foundational resources not only facilitate immediate applications in breeding and conservation but also open avenues for integrative omics approaches aimed at exploring genotype–phenotype–environment relationships.

## 5. Conclusions

This study represents the genome-wide survey of the Naozhou stock of *L. crocea*, providing crucial insights into its genetic composition and diversity. It provides valuable genomic resources, including a high-quality genome assembly, a comprehensive catalog of SSR markers, and a complete mtDNA. These resources lay a foundation for future genetic improvement programs, population monitoring, and evolutionary studies. They facilitate marker-assisted selection and genetic improvement in aquaculture, while also supporting the assessment and conservation of genetic diversity in the wild and cultured populations. Future research should focus on functional genomics and transcriptomics to explore gene expression patterns under different environmental conditions, further enhancing the understanding of the genetic mechanisms underlying adaptive traits in *L. crocea*.

## Figures and Tables

**Table 1:** Genome survey sequencing results.

Type	ReadNum	BaseCount (Gb)	Q20 (%)	Q30 (%)	GC Content (%)
raw	445,384,268	66.81	98.13	93.95	41.52
dedup	437,724,006	65.39	98.14	93.96	41.47

**Table 2:** K-mer analysis of genome characteristics of the Naozhou large yellow croaker (K = 21).

Assumed Ploidy	Haploid Genome Size (bp)	Heterozygous Ratio/Mb	Heterozygous Ratio (%)/%	Repeat Ratio (%)
2	677,786,780	0.839	0.839	22.1981

**Table 3:** The pre-assembly results of the Naozhou large yellow croaker genome.

	Contig Length (bp)	Contig Number	Scaffold Length (bp)	Scaffold Number
N90	17,745,857	23	17,745,857	23
N80	24,233,363	20	24,233,363	20
N70	25,785,482	17	25,785,482	17
N60	27,402,304	14	27,402,304	14
N50	29,070,069	12	29,070,069	12
Total length	726,425,539	-	726,425,539	-
Number (≥100 bp)	-	112	-	112
Number (≥2 kb)	-	112	-	112
Max length	34,520,400	-	34,520,400	

**Table 4:** Summary of repeated SSRs in the Naozhou large yellow croaker.

Repeats	Number	Proportion (%)
Di-	144,186	73.84
Tri-	35,019	17.93
Tetra-	11,538	5.9
Penta-	3807	1.95
Hexa-	713	0.3
Total	195,263	100

**Table 5:** The most abundant sequence categories in the genome of the Naozhou large yellow croaker.

Motif	Categories	Number	Frequency (Loci/Mb)
Di-	AC/GT	103,035	142.3447342
	AG/CT	26,685	36.86581484
	AT/AT	14,421	19.92287486
	CG/CG	45	0.062168322
Tri-	AAT/ATT	10,159	14.03484403
	AGG/CCT	6571	9.077956503
	AAC/GTT	4939	6.823318699
Tetra-	AGAT/ATCT	2459	3.397153408
	AAAT/ATTT	2200	3.039340178
	ACAG/CTGT	1444	1.994912371
Penta-	AAAAT/ATTTT	591	0.816477293
	AGAGG/CCTCT	453	0.625827773
Hexa-	AACCCT/AGGGTT	334	0.4614271

**Table 6:** Genetic diversity parameters of the Naozhou large yellow croaker at eight microsatellite loci.

Locus	Na	Ne	I	Ho	He	PIC
SSR1	9	4.8	1.7	0	0.81	0.76
SSR2	13	6.4	2.1	0.21	0.86	0.83
SSR3	5	1.54	0.70	0.88	0.36	0.32
SSR4	8	3.89	1.6	0.21	0.76	0.71
SSR5	22	10.47	2.7	0.21	0.92	0.90
SSR6	14	6.47	2.2	0.17	0.86	0.82
SSR7	16	9.14	2.4	0.29	0.91	0.88
e	4	2.4	1.0	0.75	0.60	0.51
	118	5.6	1.8	0.34	0.76	0.72
St. Dev	6.0	3.1	0.70	0.31	0.19	0.18

Note: Na—observed number of alleles; Ne—effective number of alleles; Ho—observed heterozygosity; He—expected heterozygosity; I—Shannon’s information index. PIC—polymorphism information content.

**Table 7:** Genetic diversity parameters of the Dai-qu large yellow croaker at eight microsatellite loci.

Locus	Na	Ne	I	Ho	He	PIC
SSR1	9	5.01	1.80	0.08	0.82	0.77
SSR2	9	5.2	1.82	0.21	0.83	0.78
SSR3	3	1.1	0.34	1	0.16	0.15
SSR4	9	4.2	1.70	0.42	0.78	0.73
SSR5	15	10.3	2.52	0.71	0.92	0.90
SSR6	9	5.4	1.9	0.54	0.82	0.79
SSR7	9	3.7	1.5	0.5	0.75	0.69
SSR8	5	2.9	1.2	0.79	0.67	0.59
Mean	8.5	4.7	1.6	0.53	0.72	0.68
St. Dev	3.5	2.7	0.63	0.30	0.24	0.21

Note: Na—observed number of alleles; Ne—effective number of alleles; Ho—observed heterozygosity; He—expected heterozygosity; I—Shannon’s information index. PIC—polymorphism information content.

## Data Availability

Upon reasonable request, the corresponding author will provide the data supporting the results of this study.

## Supplementary Materials

The following supporting information can be downloaded at: https://www.mdpi.com/article/10.3390/ani15172498/s1.

## References

[1] Zhang Y.Q., Guo H.Y., Liu B.S., Zhang N., Zhu K.C., Zhang D. (2024). Analysis of morphological differences in five large yellow croaker (*Larimichthys crocea*) populations. Isr. J. Aquac.-Bamidgeh.

[2] Yan L., Jiang Y., Xu Q., Ding G., Chen X., Liu M. (2022). Reproductive Dynamics of the Large Yellow Croaker *Larimichthys crocea* (Sciaenidae), A Commercially Important Fishery Species in China. Front. Mar. Sci..

[3] Zhang F., Jiang Y., Ma C., Chen W., Cheng J., Ma L. (2023). Spatial Genetic Structure and Diversity of Large Yellow Croaker (*Larimichthys crocea*) from the Southern Yellow Sea and North-Central East China Sea: Implications for Conservation and Stock Enhancement. Water.

[4] Yuan J., Lin H., Wu L., Zhuang X., Ma J., Kang B., Ding S. (2021). Resource Status and Effect of Long-Term Stock Enhancement of Large Yellow Croaker in China. Front. Mar. Sci..

[5] Cai P., Wang Z., Zhang S., Yu J. (2024). Large and Small Yellow Croakers Feeding and Living Together Make Large Yellow Croaker Population Recovery Difficult: A Guild Perspective. Biology.

[6] Wang H., Wen Z., Amenyogbe E., Jin J., Lu Y., Wang Z., Huang J. (2024). Comparative Transcriptome Analysis of Sexual Differentiation in Male and Female Gonads of Nao-Zhou Stock Large Yellow Croaker (*Larimichthys crocea*). Animals.

[7] Chen B., Bai Y., Wang J., Ke Q., Zhou Z., Zhou T., Pan Y., Wu R., Wu X., Zheng W. (2023). Population structure and genome-wide evolutionary signatures reveal putative climate-driven habitat change and local adaptation in the large yellow croaker. Mar. Life Sci. Technol..

[8] Chen X., Miao L., He Q., Ke Q., Pu F., Li N., Zhou T., Xu P. (2024). Chromosome-level genome assembly for three geographical stocks of large yellow croaker (*Larimichthys crocea*). Sci. Data.

[9] Xu G., Tian M., Zheng W. (1963). The stocks of *Pseudosciaena crocea* (Richardson). Proceedings of the 4th Plenum the Comm Fish Res the West Part the Pacific Ocean.

[10] Tian M., Xu G., Yu R. (1962). Geographic variation and population of morphological characteristics of *Pseudosciaena crocea* (Richardson). Stud. Mar. Sin..

[11] Alves S.I., Dantas C.W., Macedo D.B., Ramos R.T. (2024). What are microsatellites and how to choose the best tool: A user-friendly review of SSR and 74 SSR mining tools. Front. Genet..

[12] Das R., Arora V., Jaiswal S., Iquebal M., Angadi U., Fatma S., Singh R., Shil S., Rai A., Kumar D. (2019). *PolyMorphPredict*: A Universal Web-Tool for Rapid Polymorphic Microsatellite Marker Discovery from Whole Genome and Transcriptome Data. Front. Plant Sci..

[13] Haddrill P.R. (2021). Developments in forensic DNA analysis. Emerg. Top. Life Sci..

[14] Lu G., Luo M. (2020). Genomes of major fishes in world fisheries and aquaculture: Status, application and perspective. Aquac. Fish..

[15] Heather J.M., Chain B. (2016). The sequence of sequencers: The history of sequencing DNA. Genomics.

[16] Bian C., Huang Y., Li J., You X.X., Yi Y.H., Ge W., Shi Q. (2019). Divergence, evolution and adaptation in ray-finned fish genomes. Sci. China Life Sci..

[17] Hughes L.C., Orti G., Huang Y., Sun Y., Baldwin C.C., Thompson A.W., Arcila D., Betancur-R R., Li C., Becker L. (2018). Comprehensive phylogeny of ray-finned fishes (*Actinopterygii*) based on transcriptomic and genomic data. Proc. Natl. Acad. Sci. USA.

[18] MacKenzie S., Jentoft S. (2016). 11—Future perspective. Genomics in Aquaculture.

[19] Roest Crollius H., Weissenbach J. (2005). Fish genomics and biology. Genome Res..

[20] Lewin H.A., Robinson G.E., Kress W.J., Baker W.J., Coddington J., Crandall K.A., Durbin R., Edwards S.V., Forest F., Gilbert M.T.P. (2018). Earth BioGenome project: Sequencing life for the future of life. Proc. Natl. Acad. Sci. USA.

[21] Ke Q., Wang J., Bai Y., Zhao J., Gong J., Deng Y., Qu A., Suo N., Chen J., Zhou T. (2022). GWAS and genomic prediction revealed potential for genetic improvement of large yellow croaker adapting to high plant protein diet. Aquaculture.

[22] Shen X., Yan X., Xu J. (2018). Fatty acid profiles in muscle of large yellow croakers (*Larimichthys crocea*) can be used to distinguish between the samples of Dai-qu stock and Min-yuedong stock. Biochem. Syst. Ecol..

[23] Abdelrahman H., ElHady M., Alcivar-Warren A., Allen S., Al-Tobasei R., Bao L., Buchanan J. (2017). Aquaculture genomics, genetics and breeding in the United States: Current status, challenges, and priorities for future research. BMC Genom..

[24] Yue G., Wang L. (2017). Current status of genome sequencing and its applications in aquaculture. Aquaculture.

[25] Bernatchez L., Wellenreuther M., Araneda C., Ashton D.T., Barth J.M.I., Beacham T.D., Maes G.E., Martinsohn J.T., Miller K.M., Naish K.A. (2017). Harnessing the power of genomics to secure the future of seafood. Trends Ecol. Evol..

[26] Shen Y., Yue G. (2019). Current status of research on aquaculture genetics and genomics-information from ISGA 2018. Aquac. Fish..

[27] Liu Z.J. (2010). Next Generation Sequencing and Whole Genome Selection in Aquaculture.

[28] Jin S., Zhang X., Jia Z., Fu H., Zheng X., Sun X. (2012). Genetic linkage mapping and genetic analysis of QTL related to eye cross and eye diameter in common carp (*Cyprinus carpio* L.) using microsatellites and SNPs. Aquaculture.

[29] Xu S., Zhang H., Gao T. (2020). Comprehensive whole genome survey analyses of male and female brown-spotted flathead fish *Platycephalus* sp.1. Genomics.

[30] Choi E., Lee S.J., Jo E., Kim J., Parker S.J., Kim J., Park H. (2021). Genomic Survey and Microsatellite Marker Investigation of Patagonian Moray Cod (*Muraenolepis orangiensis*). Animals.

[31] Villarreal F., Burguener G.F., Sosa E.J., Stocchi N., Somoza G.M., Turjanski A.G., Blanco A., Viñas J. (2024). Genome sequencing and analysis of black flounder (*Paralichthys orbignyanus*) reveals new insights into Pleuronectiformes genomic size and structure. BMC Genom..

[32] Zhao X., Zheng T., Song N., Qu Y., Gao T. (2024). Whole-genome survey reveals interspecific differences in genomic characteristics and evolution of Pampus fish. Front. Mar. Sci..

[33] Kim J., Lee S., Jo E., Choi E., Cho M., Choi S., Kim J., Park H. (2022). Whole-Genome Survey and Microsatellite Marker Detection of Antarctic Crocodile Icefish, *Chionobathyscus dewitti*. Animals.

[34] Zhang Q., Hong W., Yang S., Liu M. (2011). Discussion on the division of geographic populations for the large yellow croaker (*larimichthys crocea*). Mod. Fish. Inf..

[35] Chen W., Qin H., Zhao Z., Liao J., Chen H., Jiang L., Dayananda B. (2022). The mitochondrial genome and phylogenetic analysis of Rhacophorus rhodopus. Sci. Rep..

[36] Zhang Y., Gong L., Lu X., Jiang L., Liu B., Liu L., Lü Z., Li P., Zhang X. (2020). Gene rearrangements in the mitochondrial genome of *Chiromantes eulimene* (Brachyura: Sesarmidae) and phylogenetic implications for Brachyura. Int. J. Biol. Macromol..

[37] Taanman J. (1999). The mitochondrial genome: Structure, transcription, translation and replication. Biochim. Biophys. Acta (BBA)-Bioenerg..

[38] Bagshaw A.T. (2017). Functional Mechanisms of Microsatellite DNA in Eukaryotic Genomes. Genome Biol. Evol..

[39] Chen S., Zhou Y., Chen Y., Gu J. (2018). Fastp: An ultra-fast all-in-one FASTQ preprocessor. Bioinformatics.

[40] Guichoux E., Lagache L., Wagner S., Chaumeil P., Léger P., Lepais O., Lepoittevin C., Malausa T., Revardel E., Salin F. (2011). Current trends in microsatellite genotyping. Mol. Ecol. Resour..

[41] Marçais G., Kingsford C. (2011). A fast, lock-free approach for efficient parallel counting of occurrences of k-mers. Bioinformatics.

[42] Li R., Li Y., Kristiansen K., Wang J. (2008). SOAP: Short oligonucleotide alignment program. Bioinformatics.

[43] O’Connell M., Wright J.M. (1997). Microsatellite DNA in fishes. Rev. Fish Biol. Fish..

[44] Xu S.Y., Song N., Xiao S.J., Gao T.X. (2020). Whole genome survey analysis and microsatellite motif identification of *Sebastiscus marmoratus*. Biosci. Rep..

[45] Chistiakov D.A., Hellemans B., Volckaert F.A. (2006). Microsatellites and their genomic distribution, evolution, function and applications: A review with special reference to fish genetics. Aquaculture.

[46] Marshall T.C., Slate J., BKruuk L.E., Pemberton J.M. (1998). Statistical confidence for likelihood-based paternity inference in natural populations. Mol. Ecol..

[47] Rhyker T., Jaron K.S., Schatz M.C. (2020). GenomeScope 2.0 and Smudgeplot for reference-free profiling of polyploid genomes. Nat. Commun..

[48] Yang M.Q., Athey B.D., Arabnia H.R., Sung A.H., Liu Q., Yang J.Y., Mao J., Deng Y. (2009). High-throughput next-generation sequencing technologies foster new cutting-edge computing techniques in bioinformatics. BMC Genom..

[49] Beier S., Thiel T., Münch T., Scholz U., Mascher M. (2017). MISA-web: A web server for microsatellite prediction. Bioinformatics.

[50] Greenbaum D., Hancock J.M., Zvelebil M.J. (2004). Genome-wide survey. Dictionary of Bioinformatics and Computational Biology.

[51] Xie N., Liu K., Feng X. (2016). Analysis of microsatellite information of EST sequences of *Channa argus* and *Channa maculata*. Freshw. Fish..

[52] Ji P., Zhang Y., Li C., Zhao Z., Wang J., Li J., Xu P., Sun X. (2012). High Throughput Mining and Characterization of Microsatellites from Common Carp Genome. Int. J. Mol. Sci..

[53] Li H. (2018). Minimap2: Pairwise alignment for nucleotide sequences. Bioinformatics.

[54] Jia C., Yang T., Yanagimoto T., Gao T. (2021). Comprehensive Draft Genome Analyses of Three Rockfishes (*Scorpaeniformes*, *Sebastiscus*) via Genome Survey Sequencing. Curr. Issues Mol. Biol..

[55] Prjibelski A., Antipov D., Meleshko D., Lapidus A., Korobeynikov A. (2020). Using SPAdes De Novo Assembler. Curr. Protoc. Bioinform..

[56] Fiedler L., Middendorf M., Bernt M. (2023). Fully automated annotation of mitochondrial genomes using a cluster-based approach with de Bruijn graphs. Front. Genet..

[57] Greiner S., Lehwark P., Bock R. (2019). OrganellarGenomeDRAW (OGDRAW) version 1.3.1: Expanded toolkit for the graphical visualization of organellar genomes. Nucleic Acids Res..

[58] Ziya Motalebipour E., Kafkas S., Khodaeiaminjan M., Çoban N., Gözel H. (2016). Genome survey of pistachio (*Pistacia vera* L.) by next generation sequencing: Development of novel SSR markers and genetic diversity in Pistacia species. BMC Genom..

[59] Lu M., An H., Li L. (2016). Genome Survey Sequencing for the Characterization of the Genetic Background of Rosa roxburghii Tratt and Leaf Ascorbate Metabolism Genes. PLoS ONE.

[60] Wei X., Zhou K., Zou X., Zhang X., Li Y., Luo H., Huang Y., Du X., Qin J., Chen Z. (2025). Microsatellite analyses reveal genetic diversity and population structure of *Cipangopaludina chinensis* in Guangxi, China. Aquac. Rep..

[61] Boore J.L. (1999). Animal mitochondrial genomes. Nucleic Acids Res..

[62] Yang W., Zheng J., Jia B., Chang S., Liu H., Li C., Wang G., Yang F. (2017). Research progress of microsatellite molecular markers and their application in animal genetics and breeding. Genom. Appl. Biol..

[63] Guo J., Zhang M., Wang S., Xu X., Shen Y., Li J. (2022). A high-density genetic linkage map and QTL mapping for growth related traits in grass carp (*Ctenopharyngodon idella*). Aquaculture.

[64] Fan M., Gao Y., Wu Z., Zhang Q. (2020). Linkage Map Development by EST-SSR Markers and QTL Analysis for Inflorescence and Leaf Traits in Chrysanthemum (*Chrysanthemum morifolium* Ramat.). Plants.

[65] Zhang K. (2023). Complete Genome Assembly of *Echinops punctatus* and Population Genetics Based on Microsatellites. Master’s Thesis.

[66] Liu B., Li J., Peng Y., Zhang K., Liu Q., Jin X., Zheng S., Wang Y., Gong L., Liu L. (2024). Chromosome-level genome assembly and population genomic analysis reveal evolution and local adaptation in common hairfin anchovy (*Setipinna tenuifilis*). Mol. Ecol..

[67] Barrio A.M., Lamichhaney S., Fan G., Rafati N., Pettersson M., Zhang H., Dainat J., Ekman D., Höppner M., Jern P. (2016). The genetic basis for ecological adaptation of the Atlantic herring revealed by genome sequencing. eLife.

[68] Machado A.M., Tørresen O.K., Kabeya N., Couto A., Petersen B., Felício M., Campos P.F., Fonseca E., Bandarra N., Ferraz R. (2018). “Out of the Can”: A Draft Genome Assembly, Liver Transcriptome, and Nutrigenomics of the European Sardine, *Sardina pilchardus*. Genes.

[69] Sessions S.K., Maloy S., Hughes K. (2013). Genome Size. Brenner’s Encyclopedia of Genetics.

[70] Yu J.P., Liu W., Mai C.L., Liao W.B. (2020). Genome size variation is associated with life-history traits in birds. J. Zool..

[71] Kraaijeveld K. (2010). Genome Size and Species Diversification. Evol. Biol..

[72] Zhao R., Cai S., Lu D., Li P., Xu S., Li Y. (2022). Genomic Comparison and Genetic Marker Identification of the White-Spotted Bamboo Shark *Chiloscyllium plagiosum*. Front. Mar. Sci..

[73] Zhao R.R., Xu S.Y. (2022). Whole-genome analysis microsatellite distribution characteristics of *Hemitripterus villosus*. J. Fish. Sci. China.

[74] Zhang Y.D., Wen L.T., Luo H.L., Lin Y., Du X.S., Yu Y.L., Wei Z.N., Huang Y. (2020). Genome survey development of SSRmolecular markers for Trachinotus ovatus. J. Southern Agric..

[75] Ma Y., Lou F., Yin X., Cong B., Liu S., Zhao L., Zheng L. (2022). Whole-genome survey and phylogenetic analysis of *Gadus macrocephalus*. Biosci. Rep..

[76] Chen B., Sun Z., Lou F., Song N. (2020). Genomic characteristics and profile of microsatellite primers for *Acanthogobius ommaturus* by genome survey sequencing. Biosci. Rep..

[77] Li Z., Tian C., Huang Y., Lin X., Wang Y., Jiang D., Zhu C., Chen H., Li G. (2019). A First Insight into a Draft Genome of Silver Sillago (*Sillago sihama*) via Genome Survey Sequencing. Animals.

[78] Zhu K., Zhang N., Liu B., Guo L., Guo H., Jiang S., Zhang D. (2021). A chromosome-level genome assembly of the yellowfin seabream (*Acanthopagrus latus*; Hottuyn, 1782) provides insights into its osmoregulation and sex reversal. Genomics.

[79] Huang X., Li T., Yang Y., Guo Z., Jiang J., Lin H., Fan S. (2024). Genome survey of Siganus oramin: Identification and development of genome-wide microsatellite markers. Aquac. Rep..

[80] Jo E., Cho Y.H., Lee S.J., Choi E., Kim J., Kim H., Chi Y.M., Park H. (2021). Genome survey and microsatellite motif identification of *Pogonophryne albipinna*. Biosci. Rep..

[81] Yang T., Huang X., Ning Z., Gao T. (2021). Genome-Wide Survey Reveals the Microsatellite Characteristics and Phylogenetic Relationships of *Harpadon nehereus*. Curr. Issues Mol. Biol..

[82] Carneiro Vieira M.L., Santini L., Diniz A.L., Munhoz F. (2016). Microsatellite markers: What they mean and why they are so useful. Genet. Mol. Biol..

[83] Jiang Q., Li Q., Yu H., Kong L. (2014). Genome-wide analysis of simple sequence repeats in marine animals-a comparative approach. Mar. Biotechnol..

[84] Botstein D., White R.L., Skolnick M., Davis R.W. (1980). Construction of a genetic linkage map in man using restriction fragment length polymorphisms. Am. J. Hum. Genet..

[85] Kon T., Pei L., Ichikawa R., Chen C., Wang P., Takemura I., Ye Y., Yan X., Guo B., Li W. (2021). Whole-genome resequencing of large yellow croaker (*Larimichthys crocea*) reveals the population structure and signatures of environmental adaptation. Sci. Rep..

[86] Ao J., Mu Y., Xiang L.-X., Fan D., Feng M., Zhang S., Shi Q., Zhu L.-Y., Li T., Ding Y. (2015). Genome Sequencing of the Perciform Fish *Larimichthys crocea* Provides Insights into Molecular and Genetic Mechanisms of Stress Adaptation. PLoS Genet..

[87] Xu K., Xu H., Han Z. (2018). Genome-Wide Identification of Hsp70 Genes in the Large Yellow Croaker (*Larimichthys crocea*) and Their Regulated Expression Under Cold and Heat Stress. Genes.

[88] Foulley J., Ollivier L. (2006). Estimating allelic richness and its diversity. Livest. Sci..

[89] Mao W., Xu Z., Liu Q., Li N., Liu L., Ren B., Gao T., Liu C. (2023). A Whole-Genome Survey and the Mitochondrial Genome of Acanthocepola indica Provide Insights into Its Phylogenetic Relationships in Priacanthiformes. Animals.

[90] Ojala D., Montoya J., Attardi G. (1981). tRNA punctuation model of RNA processing in human mitochondria. Nature.

[91] Hassanin A., Léger N., Deutsch J. (2005). Evidence for Multiple Reversals of Asymmetric Mutational Constraints during the Evolution of the Mitochondrial Genome of Metazoa, and Consequences for Phylogenetic Inferences. Syst. Biol..

